# Peptide Mimics of the Ribosomal P Stalk Inhibit the Activity of Ricin A Chain by Preventing Ribosome Binding

**DOI:** 10.3390/toxins10090371

**Published:** 2018-09-13

**Authors:** Xiao-Ping Li, Jennifer N. Kahn, Nilgun E. Tumer

**Affiliations:** Department of Plant Biology, School of Environmental and Biological Sciences, Rutgers, The State University of New Jersey, New Brunswick, NJ 08901-8520, USA; xpli@sebs.rutgers.edu (X.-P.L.); jennifer.nielsen.kahn@gmail.com (J.N.K.)

**Keywords:** ricin A chain, ribosomal P stalk, P protein interaction, SRL depurination, peptide inhibition

## Abstract

Ricin A chain (RTA) depurinates the sarcin/ricin loop (SRL) by interacting with the C-termini of the ribosomal P stalk. The ribosome interaction site and the active site are located on opposite faces of RTA. The interaction with P proteins allows RTA to depurinate the SRL on the ribosome at physiological pH with an extremely high activity by orienting the active site towards the SRL. Therefore, if an inhibitor disrupts RTA–ribosome interaction by binding to the ribosome binding site of RTA, it should inhibit the depurination activity. To test this model, we synthesized peptides mimicking the last 3 to 11 amino acids of P proteins and examined their interaction with wild-type RTA and ribosome binding mutants by Biacore. We measured the inhibitory activity of these peptides on RTA-mediated depurination of yeast and rat liver ribosomes. We found that the peptides interacted with the ribosome binding site of RTA and inhibited depurination activity by disrupting RTA–ribosome interactions. The shortest peptide that could interact with RTA and inhibit its activity was four amino acids in length. RTA activity was inhibited by disrupting its interaction with the P stalk without targeting the active site, establishing the ribosome binding site as a new target for inhibitor discovery.

## 1. Introduction

Ricin (E.C. 3.2.2.22), produced by the castor bean (*Ricinus communis*), belongs to a group of toxic proteins called ribosome-inactivating proteins (RIPs) that include major human pathogens, such as *Escherichia coli* and *Shigella* producing Shiga toxins (Stxs). They are classified as category B agents of national security and public health risk with potential for significant morbidity and mortality. Currently, no U.S. Food and Drug Administration-approved vaccines or therapeutics exist to protect against ricin, Stxs, or any other RIP. The RIPs cleave a universally conserved adenine from the sarcin/ricin loop (SRL) on the large rRNA, inhibiting protein synthesis and inducing cell death [1,2,3,4]. Ricin A chain (RTA) interacts with the P proteins of the ribosomal stalk to depurinate the SRL [5]. Several other RIPs, including Shiga toxins [6,7,8], also interact with the conserved C-terminal domain (CTD) of P proteins in order to access the SRL [9,10,11].

In eukaryotes, the P stalk is a pentameric protein complex composed of two P1/P2 dimers that bind to the C-terminus of the uL10 (previously P0) protein, while the N-terminal domain of uL10 anchors the stalk on the large subunit of the ribosome [12,13,14]. The P stalk and the SRL are part of the GTPase-associated center (GAC) in the large subunit [13,15]. The unique feature of all P proteins is the 11 C-terminal amino acids, which are identical in all eukaryotes and have a disordered structure [16]. The CTD of P proteins selectively recognizes translational GTPases, such as the initiation factor 5B (eIF5B) and the elongation factors eEF-2/EFG and eEF1α/EFTu, and recruits them to the SRL [17,18,19,20].

The interaction of RTA with P proteins is critical for ribosome binding, depurination of the SRL, and toxicity of RTA in the yeast *Saccharomyces cerevisiae* and in human cells [5,21]. We showed that the ribosome binding site and the active site are located on opposite faces of RTA and based on these results we proposed a molecular model for depurination of the SRL by RTA [22]. According to this model, RTA is concentrated around the ribosome by electrostatic interactions [23]. The P stalk interacts with RTA and stalk binding stimulates the catalysis of depurination by orienting the active site of RTA towards the SRL [22]. The interaction with the P proteins allows RTA to depurinate the SRL on the ribosome at physiological pH with an extremely high catalytic activity, while at this pH RTA is not active on the naked RNA [22,24]. Based on this model, we predict that if an inhibitor disrupts the interaction of RTA with the ribosome by binding to the ribosome binding site of RTA, it should be able to inhibit the depurination activity of RTA.

Until now, no inhibitor targeting the ribosome binding site of RTA has been reported. A 17-mer peptide mimicking the CTD of the human ribosomal stalk P2 protein was shown to inhibit the activity of the A1 subunit of Shiga toxin 1 (Stx1A1) in an in vitro translation assay [6]. Calmodulin-tagged peptides corresponding to the last 11 and 17 residues of the human P2 protein could pull down Stx1A1 and RTA, but not a peptide corresponding to the last 7 residues of human P2 [6]. The 11-mer peptide (P11), S_105_DDDMGFGLFD_115_, contains a negatively charged acidic motif “D_106_D_107_D_108_” at its N-terminus and a hydrophobic “F_111_GLFD_115_” motif at its C-terminus. The pull down and binding studies showed that both motifs are important for the interaction of Stx1A1 with P11 [7].

In *S. cerevisiae*, P1/P2 proteins have diverged into P1α/P2β and P1β/P2α [25]. The P1α/P2β dimer could inhibit the depurination activity of Stx1A1 and Stx2A1 on ribosomes isolated from a yeast mutant in which the binding site of the P protein dimers on the P0 protein had been deleted, suggesting that P1α/P2β can bind to the toxins and prevent them from depurinating ribosomes [8]. Although the amino acid sequences of the A1 subunit of Stx1 and Stx2 are only 21% and 20% identical to RTA, respectively, they are structurally and functionally very similar to RTA [26]. Recently, the structure of RTA together with the last six amino acids of P proteins was resolved by two different groups using different strategies (PDB IDs: 5GU4 and 5DDZ) [27,28]. Although 9-mer, 11-mer [27], or 10-mer [28] P stalk peptides were used in the studies, the structures resolved by both groups showed that only the last six residues interact with RTA. The last six residues (G_110_FGLFD_115_) bind to a hydrophobic pocket on RTA in a unique conformation. Neither group could visualize the N-terminal end of the peptide that contains the negatively charged (D_106_D_107_D_108_) motif. However, the two groups drew different conclusions. One group postulated that this motif contributes to the interaction because GST-tagged peptides, which contained the D_106_D_107_D_108_ motif, showed higher affinities for RTA than those without this motif [27]. In contrast, the other group concluded that the D_106_D_107_D_108_ motif does not contribute to the RTA interaction because they did not observe any difference in the pull-down experiments with His-tagged RTA when this motif was mutated or deleted [28]. We previously showed that arginines at the RTA/RTB interface contribute to fast electrostatic interactions with the CTD of the P proteins, indicating that the negatively charged motif plays an important role in the interaction of RTA with the ribosome [29].

Although the active site of RTA has been explored extensively as a target for antidotes, the interaction of RTA with ribosomes has not been previously examined as a potential drug target. To better understand the recognition mechanism of the P protein CTD by RTA and to define the minimal length of a peptide that can bind RTA and inhibit its activity, we measured the interaction of peptides corresponding to the last 3 to 11 amino acids of human P proteins with RTA and examined their ability to inhibit the depurination activity of RTA. We discuss the relationship between the affinity of the peptides and their inhibitory activity. Our results establish the ribosome binding site of RTA as a new target for inhibitor discovery. Since Stxs also bind to the P protein CTD to depurinate the SRL, a similar approach could be explored for Stxs.

## 2. Results

### 2.1. The Longer Peptides Have Higher Affinity for RTA than the Shorter Peptides

**Figure 1:**
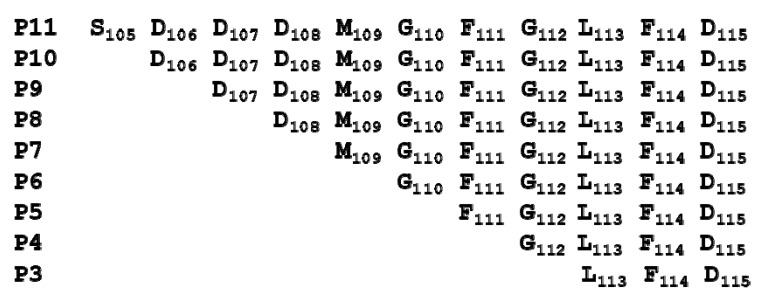
The sequence of the peptides corresponding to the C-terminal end of P proteins.

The sequences of peptides mimicking the last 11 amino acids of the P proteins are shown in Figure 1.

**Figure 2:**
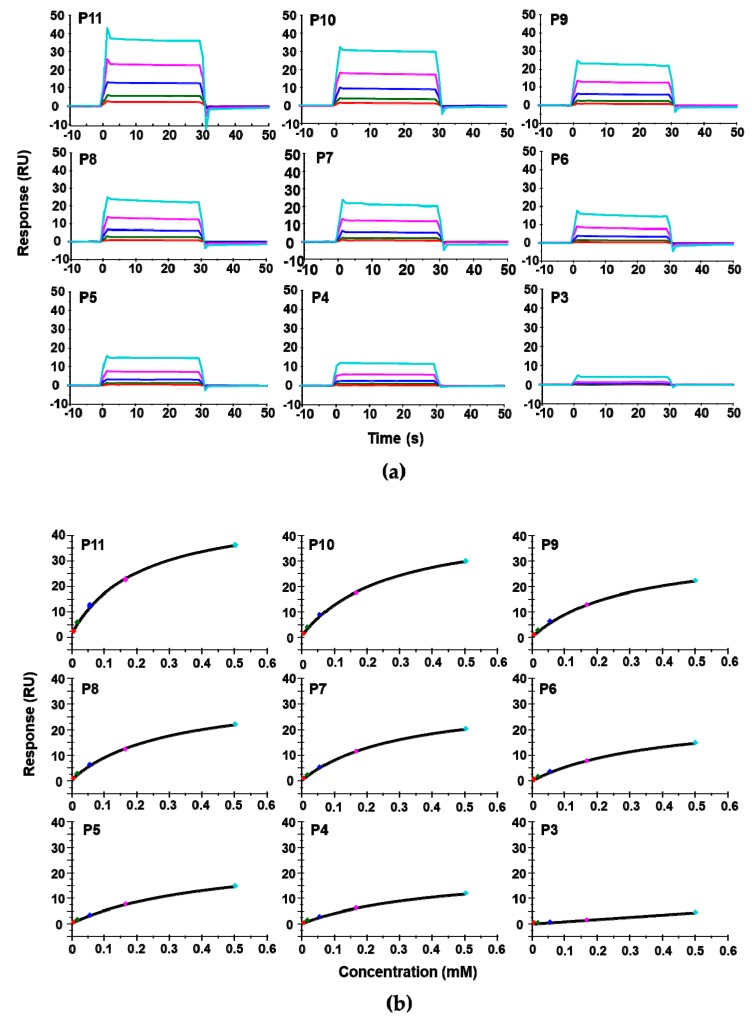
The interaction curves (**a**) and the steady state affinity fitting (**b**) of the peptide—Ricin A chain (RTA) interaction. The *K*_D_ was determined using Biacore T200. The untagged recombinant RTA was immobilized on a CM5 chip by amine coupling at about 2422 RU. The reference surface was activated and blocked. The peptides were passed over the surface at 6.2 µM (red), 18.5 µM (green), 55.6 µM (dark blue), 166.7 µM (magenta), and 500 µM (light blue).

We used surface plasmon resonance (SPR) with Biacore T200 (GE Healthcare, Marlborough, MA, USA) to measure the affinity of these peptides for RTA. Because of solubility limitations, the highest concentration of peptide measured was 0.5 mM. For direct comparison of affinity with depurination activity, depurination buffer was used as the running buffer for the Biacore analysis. The dose-dependent interaction sensorgrams are shown in Figure 2a and the fitting is shown in Figure 2b. The interaction sensorgrams of peptides with RTA showed fast on and fast off characteristics, suggesting fast association and fast dissociation, and thus relatively low affinity (Figure 2b). As the peptide concentration increased, the equilibrium binding levels increased. Due to the fast association and dissociation, equilibrium data were used to calculate the dissociation constants (*K*_D_). The *K*_D_s of the peptides are shown in Table 1. Overall, the affinity of the peptides for RTA was in the high micromolar range. The affinity of RTA for the peptides was 10^5^ times lower than its affinity for the ribosome, which is in the low nanomolar range [23]. The *K*_D_ increased from 196 µM to 451 µM as the number of amino acids decreased from 11 (P11) to 4 (P4). Deletion of the three aspartic acids (D_106_D_107_D_108_) did not decrease the affinity dramatically, as the *K*_D_ was 272 µM for P10 and around 300 µM for P9, P8, and P7. The decreased affinity was mainly due to the deletion of Asp106. The *K*_D_ did not change much when Asp107 and Asp108 were deleted. The *K*_D_ increased from 294 µM to 399 µM with the deletion of Met109 and to 497 µM when Gly110 was deleted, indicating the importance of Met109 and Gly110 in the interaction. Deleting Phe111 did not change the affinity appreciably. However, upon deleting Gly112, the affinity decreased dramatically, indicating that the peptide with only the last three amino acids of P proteins almost lost the ability to bind RTA.

### 2.2. Peptides Bind to the Ribosome Binding Site of RTA

**Figure 3:**
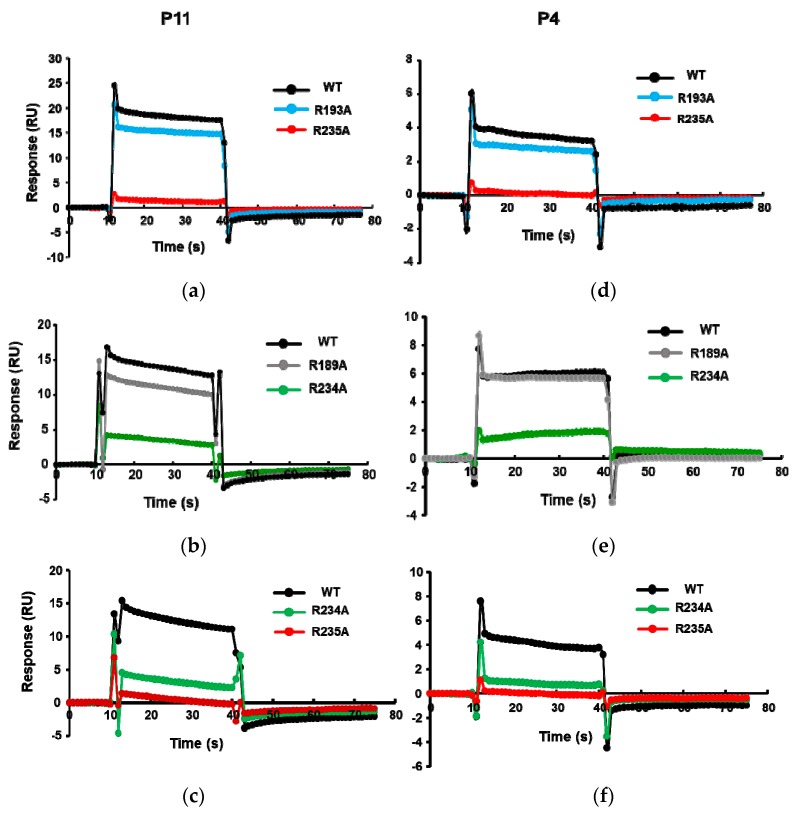
The interaction curves of P11 (**a**–**c**) and P4 (**d**–**f**) with wild-type (WT) RTA and the R193A, R235A, R189A, and R234A point mutants. The interactions were analyzed by Biacore T200 using the same conditions as in Figure 2, except N-terminally His-tagged wild-type RTA (10×His-RTA) or 10×His-tagged RTA mutants were captured on an NTA chip at around 2100 RU. Flow cell 1 (Fc1) was used as control, R235A or R234A was captured on Fc2, R193A or R189A was captured on Fc3, and wild type (WT)-RTA was captured on Fc4. The P11 or P4 were passed over the surface at a concentration of 166.7 µM. The signals were normalized to the chip density of WT-RTA.

We showed that the R189A, R193A, R234A, and R235A mutations affect the ribosome binding of RTA [29]. The order of decreased binding strength was R235A > R234A > R193A ≥ R189A [29], where the R235A mutant completely lost ribosome binding [29]. To verify the binding site of these peptides on RTA, the His-tagged RTA mutants R189A, R193A, R234A, and R235A were captured on an NTA chip and P11 and P4 were passed over the surface. As shown in Figure 3, the binding levels of P11 and P4 decreased for the Arg mutants. The relative decrease in binding levels of the Arg mutants to P11 and P4 was in the same order as the relative decrease in ribosome binding. The lowest binding was observed with R235A, followed by R234A, R193A, and R189A, indicating that the peptides bind at the ribosome binding site of RTA.

**Figure 4:**
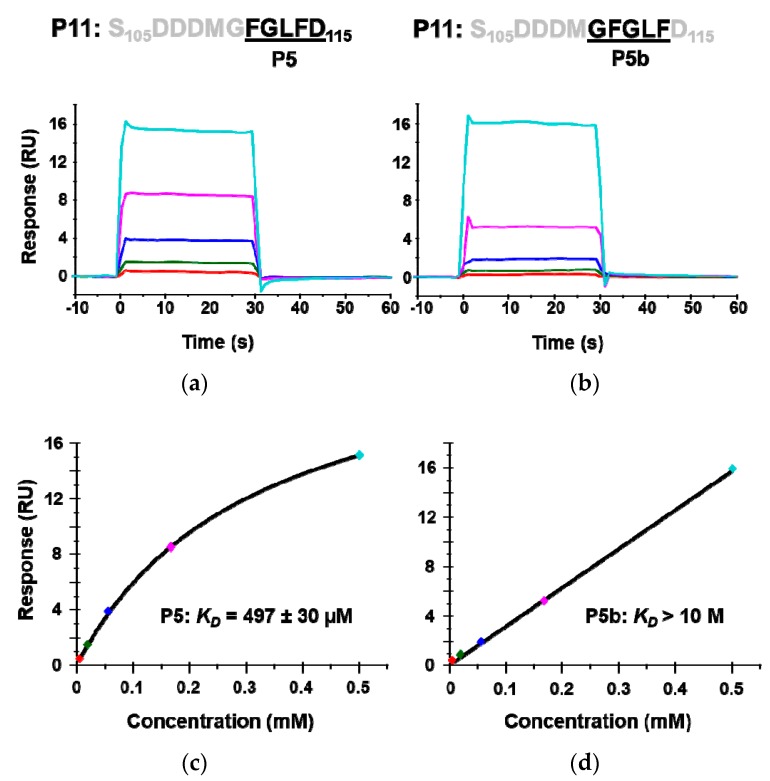
The interaction of peptides mimicking the last five amino acids (P5) of the P proteins (**a**,**c**) and the penultimate five amino acids (P5b) with RTA (**b**,**d**). The *K*_D_ was determined by Biacore T200 using the same conditions as in Figure 2. The binding sensorgrams are shown in (**a**,**b**) and the fitting is shown in (**c**,**d**).

The crystal structures of P6 with RTA have shown that Arg235 and the last aspartic acid of P proteins, Asp115, form a critical hydrogen bond. To determine if Asp115 is critical for binding RTA, a 5-mer peptide P5b (G_110_FGLF_114_) in which Asp115 was deleted was synthesized and its interaction with RTA was compared to P5 (F_111_GLFD_115_). The calculated *K*_D_ of P5b was over 20 times higher than that of P5, indicating that the last aspartic acid (D_115_) is critical for the interaction with RTA (Figure 4). These results provide further evidence that the peptides interact with the ribosome binding site of RTA.

### 2.3. Peptides Compete with Ribosomes for Binding to RTA

**Figure 5:**
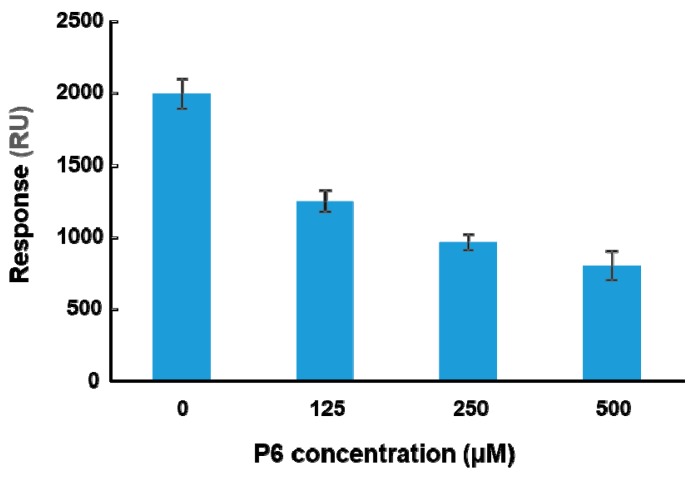
P6 competes with the ribosome for binding to RTA. The A-B-A capability of Biacore 8K was used for the competition analysis. RTA was immobilized on a CM5 chip at 4000 RU by amine coupling. The P6 was passed over the RTA at indicated concentrations for 1 min and then yeast ribosomes (20 nM) were injected over the surface together with the same concentrations of P6 for another 2 min at a flow rate of 30 µL per min. The ribosome binding levels were determined at 5 s before the end of the injection. The surface was regenerated by three one-minute injections of 2 M NaCl and one injection of running buffer with 2% DMSO. The data are expressed as average ± SD from four replicates.

To determine if peptide binding at the ribosome binding site of RTA can affect binding of RTA to ribosomes, a peptide-ribosome competition assay was conducted using the A-B-A injection capability of Biacore 8K. The molecular weight of the yeast ribosome is about 3000 times larger than that of the peptides. The affinity of RTA for the ribosome is in the nanomolar range [23]. The affinity of peptides for RTA is in the high micromolar range (Table 1) and about 10^5^-fold lower than the affinity for the ribosome. Since the structure of the last six amino acids of P proteins with RTA has been resolved, P6 was chosen for the competition assay. RTA was immobilized on a CM5 chip by amine coupling. The surface was first blocked by P6 at three different concentrations (125, 250, and 500 µM) for one minute. Ribosomes (20 nM) were mixed with the same three concentrations of P6 and passed over the surface for two minutes. The ribosome binding levels at the end of each injection were plotted against the concentration of P6. As shown in Figure 5, ribosome binding decreased as the concentration of P6 increased, indicating that P6 competed with ribosomes for binding to RTA in a dose-dependent manner. However, 10^4^-fold higher concentration of P6 was needed compared to the ribosome and the inhibition did not reach 100%.

### 2.4. Peptides Inhibit the Depurination Activity of RTA

**Figure 6:**
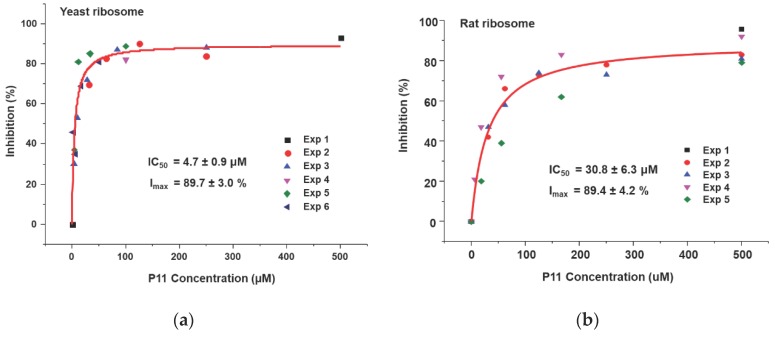
Inhibition of depurination activity of RTA by P11. The depurination levels were determined by qRT-PCR. (**a**) Yeast ribosomes were used at 60 nM and RTA was used at 1.0 nM. (**b**) Rat liver ribosomes were used at 60 nM and RTA was used at 0.2 nM. Different concentrations of P11 and RTA were mixed first and the reaction was started by adding ribosomes. The reaction was incubated at the room temperature for 5 min and was stopped by adding 2×RNA extraction buffer. The RNA was purified and the depurination levels were determined by qRT-PCR. The depurination level of the reaction without toxin was set as 100%. The depurination levels were calculated and plotted as percent of no toxin control. Experiments were conducted four to six times and the data were fit with the Michaelis–Menten equation using Origin Pro 9.1. IC_50_: the half maximal inhibitory concentration. I_max_: maximal inhibition.

We showed that the peptides bind to the ribosome binding site of RTA and affect the interaction of RTA with the ribosome. Based on our ribosome depurination model, if ribosome binding of RTA is reduced then depurination of the SRL on the ribosome should be affected. To test this model, we examined the ability of the peptides to inhibit the depurination activity of RTA on yeast and rat liver ribosomes. Both ribosomes were used since RTA depurinates rat liver ribosomes at a much higher rate than yeast ribosomes [30]. Different concentrations of peptides were incubated with RTA first, and then ribosomes were added to start the depurination reaction. After 5 min at room temperature, the reaction was stopped and the level of ribosome depurination was measured by qRT-PCR [31]. The percent depurination was plotted against the concentration of the peptide (Figure 6). The IC_50_ values were obtained by fitting the inhibition curves with the Michaelis–Menten equation using the Origin Pro 9.1 software (OriginLab, Northampton, MA, USA). The inhibition of depurination of yeast (Figure 6a) and rat liver (Figure 6b) ribosomes by P11 are shown. As peptide concentrations increased, the percentage of inhibition increased, and percent inhibition reached 90% for both ribosomes. The IC_50_ of P11 was 4.7 µM for yeast ribosomes and 31 µM for rat liver ribosomes (Table 2). The IC_50_ values of P10 to P3 were measured using the same method for yeast (Figure S1) and rat liver (Figure S2) ribosomes and data are shown in Table 2. The IC_50_ values for yeast ribosomes increased from 7.9 µM to 15 µM when D_106_ was deleted and to 23 µM and 34 µM when D_107_ and D_108_ were deleted, respectively. Similarly, the IC_50_ values for rat ribosomes increased from 83 µM to 142 µM when D_106_ was deleted and to 267 µM when D_107_ was deleted. These results demonstrated that the D_106_D_107_D_108_ motif is important for inhibition of the depurination activity of RTA. The IC_50_ values were about 6 to 10 times higher for rat ribosomes than yeast ribosomes, possibly because RTA depurinates rat ribosomes at a higher rate than yeast ribosomes [30]. We could not measure IC_50_ for P7 to P4 for rat ribosomes because the peptide concentrations needed were prohibitive and limited by solubility. We could not detect any inhibition activity for P3 and P5b at the highest concentration measured (500 µM). The IC_50_ values correlated with the RTA interaction results (Table 1) and indicated that inhibition of ribosome binding by peptides with even a low affinity could lead to inhibition of the depurination activity of RTA.

## 3. Discussion

The active site of RTA has been explored extensively as a potential target for antidotes against depurination [32]. However, since SRL is the substrate of RTA, the active site is large and mostly polar and therefore small molecule inhibitor screens have yielded few potential inhibitors with low affinity [32]. Although inhibitors showed activity in enzymatic tests, they failed to protect cells or animals against ricin challenge. Only one small molecule has been shown to have activity in protecting mice against ricin challenge by blocking the retrograde trafficking of ricin [33]. To address this barrier and to establish a starting point for inhibitor discovery, we established the ribosome binding site of RTA as a new target and identified the shortest length of a peptide that can bind to the ribosome binding site of RTA and inhibit its activity.

The C-terminal ends of the ribosomal stalk P proteins interact with a small well-defined hydrophobic pocket on the face of RTA opposite to the active site [27,28]. We show here that peptides derived from the conserved CTD of P proteins can disrupt RTA–ribosome interactions. The longest peptide tested was P11 because this is the smallest peptide reported to inhibit the activity of Stx1 [6]. Since the C-terminal end of this peptide is critical for RTA interaction [27,28], we deleted one amino acid at a time starting from the N-terminal end. We found that the longer peptides had higher affinity and inhibitory activity compared to the shorter peptides. The shortest peptide that could interact with RTA and inhibit its activity corresponded to the last four amino acids of P proteins.

A conserved D_106_D_107_D_108_ motif at the N-terminal end of P11 has been shown to be critical for the interaction with trichosanthin (TCS) [34]. In the structure of the TCS–P11 complex, Asp108 of P11 interacted with Lys173 of TCS via salt bridges, while Asp106 of P11 formed hydrogen bonds with Gln169 of TCS [35]. However, in the two structures of this peptide with RTA, the D_106_D_107_D_108_ residues were not observed [27,28]. The substitution of Asp108 and Asp106 residues in P2 with alanine abolished the interaction between P2 and TCS [35]. However, these mutations did not affect the interaction of P2 with RTA [28]. Based on these results, Fan et al. concluded that the conserved D_106_D_107_D_108_M_109_ motif of P2 is not involved in the interaction with RTA and only hydrophobic interactions and hydrogen bonds contribute to the interaction [28]. In contrast, Shi et al. showed that although the D_106_D_107_D_108_ motif was not observed in the structure, the binding affinity of RTA measured by isothermal calorimetry (ITC) was lower when this motif was not present on the peptide than when this motif was present [27].

To address the role of the negatively charged motif at the P protein CTD, we deleted these residues one at a time. Our results indicate that individual deletion of Asp_107_ and Asp_108_ in the D_106_D_107_D_108_ motif had negligible effect on affinity of the peptide for RTA, while deletion of Asp_106_ had a small effect. This may explain why the GST-tagged P2 variants containing Asp to Ala mutations in this motif interacted with RTA in the pull-down experiments [28]. When affinity was measured directly using Biacore T200, we observed a small decrease in *K*_D_ from 272 µM to about 300 µM when Asp_106_ was deleted. However, the IC_50_ increased 2-fold upon deletion of Asp_106_. The IC_50_ increased further 2-fold when Asp_107_ was deleted (Table 2). This data is consistent with our previous study [29] where mutation of positively charged arginines on RTA led to a significant increase in *K*_m_ toward ribosomes without affecting the *K*_m_ or *k*_cat_ towards an RNA mimic of the SRL, indicating that electrostatic contacts contribute to the interaction of RTA with the ribosome [29]. We showed that arginines are critical for maintaining the fast association and dissociation rates of the interaction with the CTD of P proteins [29]. We proposed that these arginines form a positively charged patch on the surface of RTA and interact with the negatively charged D_106_D_107_D_108_ motif at the CTD of the P proteins to facilitate the interaction of RTA with the P stalk to allow depurination of the SRL [29]. The results presented here provide direct evidence that the D_106_D_107_D_108_ motif is important for ribosome anchoring of RTA for depurination of the SRL.

The qRT-PCR based depurination assay, which directly measures the catalytic activity of RTA on ribosomes showed demonstrable impact on RTAs ability to depurinate ribosomes in a manner which correlated with ribosome binding [36]. Comparison of the binding affinity and the IC_50_ of RTA for the peptides indicated that the IC_50_ values were lower than the *K*_D_ values. The P11 with 200 µM *K*_D_ was able to achieve 50% inhibition of RTA activity at 5 µM, which is about 40 times lower than the *K*_D_. For the shorter peptides, such as P5 and P4, the IC_50_ (~100 µM) values were about 5 times lower than the *K*_D_ (~500 µM) values. Although peptides do not bind RTA tightly, as indicated by the high *K*_D_ values, they inhibit the activity of RTA, as indicated by the relatively low IC_50_ values. This difference may be because of allosteric binding sites, where peptides are binding in a location separate from the active site. Thus, peptides do not compete with binding of the active site of RTA to the SRL, but compete with binding of RTA to the P stalk. Our results indicate that inhibition of depurination activity involves both electrostatic and hydrophobic surfaces on the P protein peptide. Electrostatic interactions are critical to maintain the high association and dissociation rates of RTA with the P proteins on the ribosome [28]. Even low affinity binding to the P protein peptide led to a high level of inhibition of depurination by RTA, suggesting that the ribosome binding site is a potentially valuable target distinct from the active site. However, due to the low affinity of the peptides for RTA in combination with the high catalytic efficiency of RTA the therapeutic potential of the peptides used in this study is limited and is not a claim of this paper. They need to be optimized into higher affinity ligands. We establish the ribosome binding site as a potential new target for inhibitor discovery as a proof of concept. Since P protein CTD is the binding site of several RIPs, including the Stxs, inhibitors targeting the ribosome interaction site of RTA could be effective against the Stxs.

The structural analysis showed that the binding between P10 and RTA is mediated by hydrophobic interactions. The Phe111, Leu 113, and Phe114 residues are inserted into a hydrophobic pocket and the Phe114 and Asp115 residues form hydrogen bonds with Arg235 of RTA [27,28]. Consistent with the structural analysis, our results indicated that P5 and P4 showed similar affinity and inhibition activity for RTA (Table 1 and Table 2). The binding and depurination inhibition results of P5 over P5b (Figure 4) confirmed that Asp115 plays an important role in the interaction. However, P3 containing both Phe114 and Asp115 only bound very weakly to RTA and could not inhibit RTA activity. Although the conformation of TCS bound to P11 differed from the structure of RTA, both RIPs recognized the Leu113 and Phe114 motif [28]. This LF motif is conserved in both eukaryotic and archaeal ribosomal stalk proteins and has been shown to be necessary for binding of translational GTPases to the stalk proteins [37,38]. Our results suggest that RTA binding to P3 is substantially reduced when Gly112 is deleted possibly because Gly112 accommodates the required backbone to facilitate the insertion of Phe111, Leu113, and Phe114 into the hydrophobic pocket on RTA [28]. The structural analysis showed that the C-terminal sequences of P proteins do not form a stable structure in solution in a ligand-free state [16], but appear as an α-helix upon binding to the hydrophobic pocket of RTA [27,28]. Since 3.6 amino acids are the minimum length needed to form an α-helix, the last three amino acids may not interact well with RTA because they cannot form an α-helix even though they contain all the critical side chains for the interaction. These results indicate that the minimal length of P protein CTD required for binding to RTA and inhibiting its activity is four amino acids.

The C-terminal sequences of the stalk proteins can adapt diverse conformations in order to bind distinct ligands specifically [16]. The P11 appeared as a type II β-turn upon binding to TCS [35]. However, the last six amino acids of P11 formed an α-helix when bound to RTA [27,28]. Similarly, the CTD of archaeal P1 (aP1) formed a β-turn and a 3_10_-helix when bound to eIF5B [20]. In contrast, the CTD of aP1 bound to eIF1A formed a long extended α-helix [37]. The aP1 bound to a hydrophobic pocket on the surface of eIF5B, which is present on the opposite side of the GTP/GDP binding site [20]. It was suggested that the stalk/eIF5B interaction contributes to the recruitment of the GTP binding site of eIF5B to the SRL [20]. Our results indicate that RTA interacts with rapid on and off rates and with low affinity with peptides mimicking the C-terminal sequence of the P proteins. The A1 subunit of Stx1 was also shown to interact with low affinity with P11 [7]. RIPs and translational GTPases may interact with low affinity with the stalk proteins to properly orient their active site and the GTP binding site, respectively, towards the SRL. Thus, our RTA–ribosome interaction model, which proposes that the interaction with the stalk stimulates the catalysis of depurination by orienting the active site of RTA towards the SRL [22], may be applicable to other RIPs and the translational GTPases that interact with the stalk.

## 4. Materials and Methods

### 4.1. Peptide Synthesis

Peptides were synthesized by GenScript (Piscataway, NJ, USA) at purity higher than 98%. The purity and the sequence accuracy were confirmed by HPLC and mass spectrometry.

### 4.2. RTA Purification

Wild-type untagged RTA was purified using a previously published method [30]. His-tagged wild-type RTA was purified as previously described [22]. His-tagged RTA mutants were purified by the Northeast Biodefense Center Protein Expression Core. Yeast and rat liver ribosomes were purified using a previously published method [5].

### 4.3. Peptide–RTA Interaction

The peptide–RTA interaction was measured by surface plasmon resonance (SPR) using Biacore T200. The untagged RTA was immobilized on a CM5 chip at 2000 to 3000 RU by amine coupling. The reference channel was blocked as the active channel. The peptides were passed over the surface at 6.2, 18.5, 55.6, 166.7, and 500 µM at a flow rate of 30 µL/min. The running buffer was 10 mM Tris-HCl pH7.5, 60 mM KCl, 10 mM MgOAc, and 0.5% DMSO. The affinity was obtained by fitting the binding data using Biacore T200 Evaluation Software version 3.0.

The peptide–RTA mutant interactions were measured using Biacore T200. The 10×His-tagged wild-type RTA or the 10×His-tagged RTA mutants were captured on an NTA chip to around 2000 RU. The blank surface without Ni^+^ was used as the reference. The peptides were passed over the surface at 30 µL/min. The running buffer was the same as the untagged RTA. The binding signals were normalized for the differences in surface density.

### 4.4. Peptide Competition Assays

The peptide competition assays were conducted using Biacore 8K (GE Healthcare, Marlborough, MA, USA). The untagged RTA was immobilized on a CM5 chip to around 4000 RU in flow cell 2 for all eight channels by amine coupling. The flow cell 1 was activated and blocked. The A-B-A injection was used. The surface was first injected for 1 minute with peptide P6 at 0, 125, 250, and 500 µM. Then, yeast ribosomes (20 nM) mixed with the same concentrations of P6 were injected on the surface for 2 min. Ribosome disassociation was allowed for another 2 min. The flow rate was 30 µL/min. The surface was regenerated by three one-minute injections of 2 M NaCl and one 1 min injection of running buffer containing 2% DMSO. The running buffer was the same as peptide–RTA interaction buffer with 0.005% of surfactant P20. The binding levels of ribosome at “A-B-A binding later” were compared.

### 4.5. Inhibition of RTA Depurination

The inhibition of depurination activity was measured by qRT-PCR using the untagged RTA [31]. The final RTA concentration was 1.0 nM for yeast ribosomes and 0.2 nM for rat liver ribosomes with both ribosome concentrations at 60 nM. The same buffer used to examine the interaction of RTA with peptides was used. The final peptide concentrations varied dependent on the level of inhibition by the peptides. RTA was mixed with the peptides at room temperature for several minutes. The ribosomes were added to start the reaction. The depurination reaction was incubated at 25 °C for 5 min and was stopped by adding an equal volume of RNA extraction buffer. RNA was purified from the depurinated ribosomes using a previously published method [22]. The percentage of depurination was determined by qRT-PCR [31]. In each set of measurements, the ribosome and peptide mixture without toxin was used as 100% and the mixture with ribosome and toxin but without the peptide was used as 0%. The percentage of inhibition was plotted against the peptide concentration and the IC_50_ was calculated by fitting the data with the Michaelis–Menten function using Origin Pro 9.1.

## Figures and Tables

**Table 1:** The affinity of peptides for RTA.

Peptides	Sequence	MW	*K_D_* (µM) *
P11	S_105_DDDMGFGLFD_115_	1218.25	196 ± 17
P10	D_106_DDMGFGLFD_115_	1131.18	272 ± 6
P9	D_107_DMGFGLFD_115_	1016.09	309 ± 7
P8	D_108_MGFGLFD_115_	901.00	299 ± 5
P7	M_109_GFGLFD_115_	785.91	294 ± 47
P6	G_110_FGLFD_115_	654.71	399 ± 20
P5	F_111_GLFD_115_	597.66	497 ± 30
P4	G_112_LFD_115_	450.49	451 ± 17
P3	L_113_FD_115_	393.44	>10 mM

* Data were obtained from the fitting shown in Figure 1 and shown as average ± SD of three to four replicates. MW: molecular weight. ***K_D_***: the equilibrium dissociation constant.

**Table 2:** Inhibition of depurination activity of RTA by peptides.

Peptide	Sequence	Yeast Ribosome IC_50_ (µM) *	Rat Ribosome IC_50_ (µM) *
P11	S_105_DDDMGFGLFD_115_	4.7 ± 0.9	31 ± 6.3
P10	D_106_DDMGFGLFD_115_	7.9 ± 1.7	83 ± 18
P9	D_107_DMGFGLFD_115_	15 ± 1.5	142 ± 61
P8	D_108_MGFGLFD_115_	23 ± 4.4	267 ± 80
P7	M_109_GFGLFD_115_	34 ± 9.5	NA
P6	G_110_FGLFD_115_	63 ± 13	NA
P5	F_111_GLFD_115_	121 ± 44	NA
P4	G_112_LFD_115_	102 ± 45	NA

* The IC_50_ values were determined using the method shown in Figure 6 and Figures S1 and S2. Data are from the fitting results. NA: not analyzed.

## Supplementary Materials

The following are available online at http://www.mdpi.com/2072-6651/10/9/371/s1, Figure S1: Inhibition of depurination activity of RTA on yeast ribosomes by peptides P10–P3. The IC_50_ values were determined as in Figure 6 and are shown in Table 2. Figure S2: Inhibition of depurination activity of RTA on rat liver ribosomes by peptides P10, P9, and P8. The IC_50_ values were determined as in Figure 6 and are shown in Table 2.
