# Supplementary material for: Peptide Mimics of the Ribosomal P Stalk Inhibit the Activity of Ricin A Chain by Preventing Ribosome Binding
Source: Toxins (Basel). 2018 Sep 13;10(9):371. doi: 10.3390/toxins10090371 (PMC6162817; doi:10.3390/toxins10090371)
Supplement: Supplementary file 1 [file toxins-10-00371-s001.pdf]

Supplementary Materials: Peptide Mimics of the Ribosomal P Stalk Inhibit the Activity of Ricin A Chain by Preventing Ribosome Binding

Xiao-Ping Li, Jennifer N. Kahn and Nilgun E. Tumer

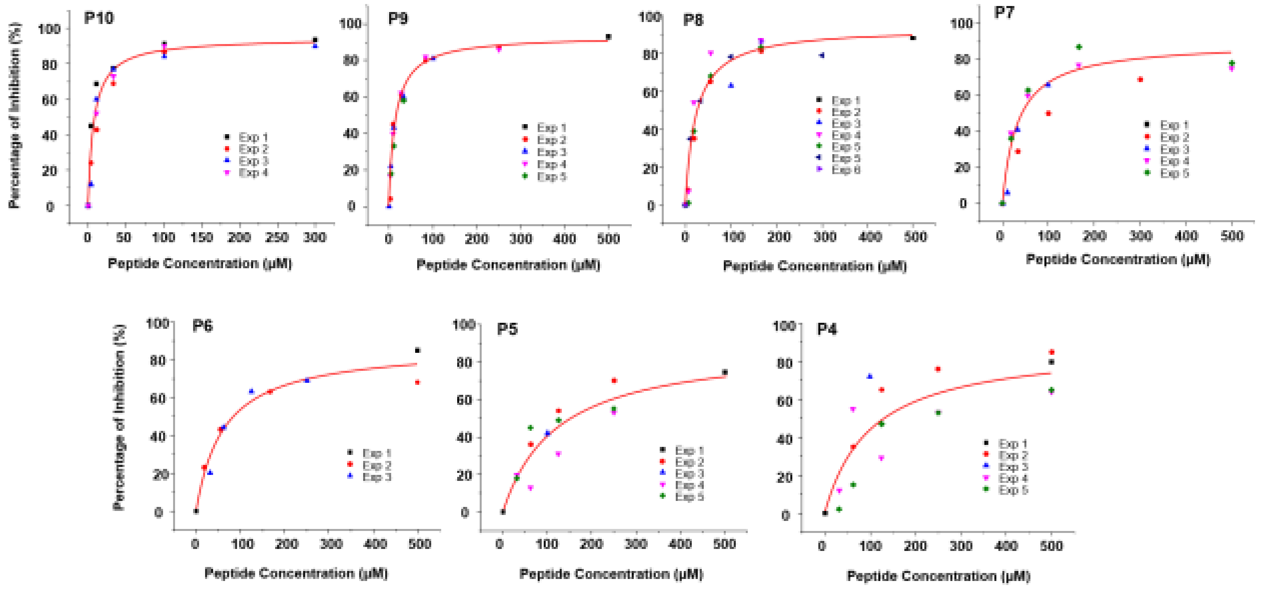

Figure S1. Inhibition of depurination activity of RTA on yeast ribosomes by peptides P10–P3. The IC_{50} values were determined as in Figure 6 and are shown in Table 2.

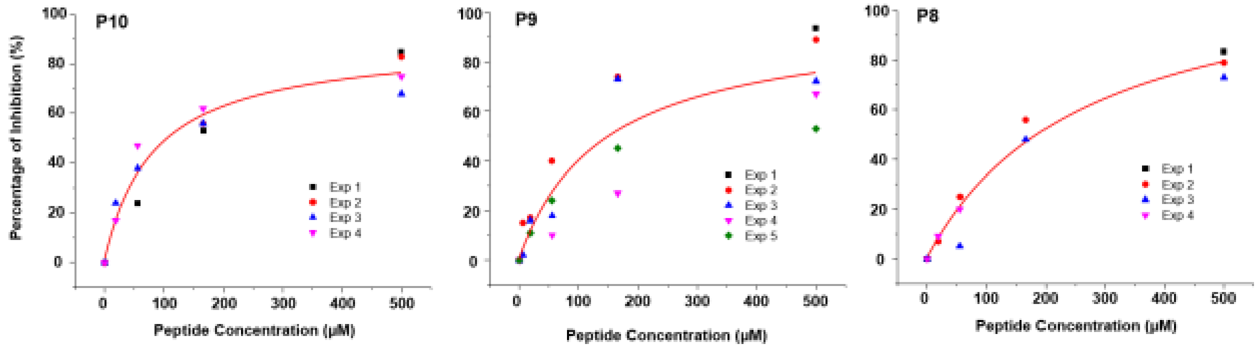

Figure S2. Inhibition of depurination activity of RTA on rat liver ribosomes by peptides P10, P9, and P8. The IC_{50} values were determined as in Figure 6 and are shown in Table 2.
